# Our Correspondence

**Published:** 1871-10

**Authors:** 


### Our Correspondence.

HUBBARDSTON, Iona Co., Mich.
Aug. 24, 1871.

THAD S. UP DEGRAFF:

*My Dear Sir:*—Enclosed is a circular letter, which explains itself. Give 'em fits in the BISTOURY. It was sent to my wife, and in her indignity she knew of no better way to have justice done all concerned, than to send it to you. It's at your disposal.

I am, truly yours, &c.,
E. P., M. D.

The circular referred to is entitled, "Fifteen Minutes' Private Conversation with Married Ladies, by one of Their Number;" and purports to be issued by a Mrs. H. Metzger, from a little town in Pennsylvania.

It is a most disgusting affair, pandering to the tastes of the lowest order of intellect,—a catchpenny concern, intended to find customers for articles, even if they *could* possibly accomplish what they are represented to do, would not be found in the possession of *any one* laying the least claim to intelligence or refinement. The country is flooded by such abominable literature, and strange to say, there is no law at present in existence by which such traffic can be suppressed.

RAYVILLE, Ray Co., Mo.,
May 1, '71.

THAD S. UP DEGRAFF, M. D.:

*Dear Sir:*—Your BISTOURY is received, read and well liked. You are doing a good work. If you will send me a few specimen copies of the BISTOURY, I think I can easily obtain a club for your valuable magazine. You will greatly oblige me by sending me a formula for scrofulous ophthalmia, also one for opacity of cornea, in your next issue.

Accept my best wishes for your continued success, and allow me to subscribe myself,

Yours truly, J. H. F., M. D.

To furnish you with a formula for "scrofulous ophthalmia," would necessitate an essay upon keratitis in general. The treatment must be adapted to the particular case. Read Wells, or Stellwag on the Eye.

For opacity of the cornea, unaccompanied by inflammation, the following will be found valuable for general use:—

℞
Pot. iodidum,
Yellow oxide of mercury, āā gr. j;
Adeps, ℥ iss. M.

Apply a particle, as large as a pin head, to the opacity, once daily.

---

LIMA, N. Y., Sept. 6, 1871.

DR. UP DEGRAFF:—

*Dear Sir:*—I have a sister blind from *cataract*. A physician in Rochester is doctoring her for her blood, and says he can so remove the cataract. Can he do it? Your article on cataract in the January number of the BISTOURY, leads me to suppose that nothing short of an operation will reach such a case.

R——.

When your physician in Rochester, or elsewhere, succeeds in removing *cataract* by "doctoring the blood," let us know—we would like to learn his art. Ask him if he can amputate a leg in the same manner!

---

WHEELING, W. Va., Aug. 20, 1871.

DR. THAD S. UP DEGRAFF:

*Sir:*—I see the inclosed notice going the rounds of our southern papers. What does it mean? R. M. M.

AVOID QUACKS!—A victim of early indiscretion, causing nervous debility, premature decay, &c., having tried in vain every advertised remedy, has discovered a simple means of self-cure, which he will send free to his fellow-sufferers. J. H. REEVES, 78 Nassau street, New York.

It means that, when you send for his *free* formula, you will receive one that no druggist in your, or any other city, can prepare. Therefore, you will be obliged, if you are green enough, to send to the swindling humbug for his medicine, which his circular will inform you can be had of him "ready prepared," for a sum varying from $3 to $10. We have exposed these swindlers repeatedly, in former numbers of the BISTOURY.

---

JACKSONVILLE, Fla., July 30, 1871.

THAD S. UP DEGRAFF, M. D.,:

*Dear Sir:*—My physician sent me here last winter to cure me of *amaurosis*, saying that when my general health was restored, my vision would be likewise. I have remained here ever since and find my vision is gradually growing worse, until now it is with difficulty that I can see to walk. I wish you would advise me what to do, and send me a prescription for amaurosis, for which I will gladly pay.

MRS. W. A.

You ask of us more than we can give. You have wasted too much time already. Go to some skillful oculist and have him make a *proper* diagnosis of your case. Perhaps he can benefit you,—perhaps not. Do not waste more time in waiting for your vision to improve—it won't do it. *Amaurosis* means nothing in this day of ophthalmic science; you may have one of a hundred different forms of disease of the retina, or of the humors of the eye.

---

TROY, Pa., Sept. 5, 1871.

DR. UP DEGRAFF:

*Dear Sir:*—Please advise me what to do with my daughter, sixteen years old, who is gradually loosing her vision. She has been studying too hard at school lately, so that her eyes become painful, look bloodshot, and vision quite indistinct, when she attempts to read. She does not want to give up her school, yet can persue her studies only with great difficulty, with her eyes as they are at present. What should she do? M——.

Quit school, and all employment requiring close application of the eyes, at once. Must not look into a book or paper of any sort. Apply to your family physician for suitable means for invigorating her general health. Bathe her eyes several times during the day in cold water. If she does not speedily improve, consult a competent oculist before it is too late.

---

SUSQUEHANNA DEPOT, Pa., Aug. 21, 1871.

DR. UP DEGRAFF:

We have a little child one month old which has a club foot. Its foot turns in, so that if it stood upon its feet, it would be resting upon the outer edge of its right foot. Can it be operated upon so young, and what would you advise us to do? J. S. R.

You can have the foot straightened without an operation, if you attend to it at once. A suitable apparatus can be fitted to it, which will restore the foot to its natural position in a few months' time.

---

OLNEY, Ills., July 18, 1871.

THAD S. UP DEGRAFF, M. D.:

*Dear Sir:*—Have you any of that cancer plant, called *conderango?* My mother has what the doctors call a cancer in her breast. It has not yet broken out, and we thought she might be cured if we could get some of that medicine. If you have any, let me know what it costs and what you think of it, and I will send your price immediately. P. F.

We have no conderango, and don't want any, —neither do you. It is an ingeniously advertised humbug, and is worse than useless. The sort of conderango your mother wants is a skillful surgeon with a good knife. If he can't cure the malady, nothing yet discovered can.

---

BUFFALO, N. Y., Sept. 6, 1871.

DR. UP DEGRAFF:

Can you send me a formula for hæmorrhoids? Am much troubled with constipation, and at such times the tumors protrude, causing me much suffering. M——.

Regulate your diet so as to remove constipation. This can be easily done: Eat, for breakfast, brown bread and oat meal porridge—nothing else. Dinner, a full meal of meats and vegetables. Supper, nothing but boiled, cracked wheat, with milk or sugar. Go to stool at a *regular hour* every morning,—whether you feel like it or not,—go! Anoint the tumors with ten grains of tannin, dissolved in two ounces of pure glycerine, after every movement of the bowels. Obey directions, and you can soon cure you hæmorrhoids.

---

"The New York Case Prize Co," is respectfully informed that we do not publish foreign advertisements without cash payment in advance, and that swindling operations are not received at *any* price.

---

Dr. S. D., *Unadilla, Mich.*, did not read our article on Diagnosis, in last number of BISTOURY, else he would have discovered that we *did* make some comments upon Mr. Pratt's article upon Medical Reform. In this connection, we desire to have it understood that we do not hold ourself responsible for the opinions of our contributors, neither must we be understood to agree with them in *all* that is expressed in their writings.

---

Dr. L., *Macon Ga.*—We prefer Wells to Stellwag, on the Eye. Either will answer you purpose. Hamilton on fractures is the best American work on that subject.

---

Dr. M, *Poughkeepsie, N. Y.*—Babcock's silver uterine supporter is far superior to the one you mention. It is considered the best in use—is more costly, but worth more. You will find the Dr.'s address in the BISTOURY.

---

Dr. C., *Cumberland, O.*—A good lithotrite will cost you from $30 to $75. Address Geo. Tiemann & Co., N. Y., for descriptions.

---

Dr. ——, *Cleveland, O.*—Your article will have to be re-written. We have not the time nor inclination to do it. You can do better,—try again.

---

Mrs. J. S., *Hornellsville, N. Y.*, is informed that we know nothing of the traveling doctor she mentions. If there is no regular, resident physician in your place, and if you desire to be humbugged, employ a traveling doctor.

---

### Protection for Wet Weather.

High rubber boots are very nice for children in weather that is bright over head but wet under foot. I think that one pair serves my little boy through three wet seasons—two springs and one fall—and it is a great pleasure and some profit to him to wade out into the vin-lakes, vin-oceans, and vin-rivers made by rain or melted snow. "Vin" in his "Tench" language, means "dry-away-soon," I am informed.

Rubber boots are indispensable for women, who are obliged to be out in all weathers and who wish to preserve good health. I often wonder why we who love the woods and fields do not provide ourselves with costumes suitable for rambling about comfortably. I remember that Mr. Beecher, (in one of the first series of "Star Papers," I believe,) recommended the Bloomer for such occasions. So did Grace Greenwood in her early Greenwood Leaves. But that comfortable costume has fallen into such disrepute among persons who fancy that they already know and apply the laws of beauty in regard to woman's dress, that it requires great courage for a sensitive woman to "face a frowning world," even on a stormy day, in a dress that protects her person without wearying her in both body and mind by the constant care she is obliged to give.—*Faith Rochester*.

---

Sleeping rooms should be constructed with great care and strict regard to ventilation, as one-third of human life is spent in them.

